# Use of Antihypertensive Drugs During Preeclampsia

**DOI:** 10.3389/fcvm.2018.00050

**Published:** 2018-05-29

**Authors:** Obinnaya Odigboegwu, Lu J. Pan, Piyali Chatterjee

**Affiliations:** Department of Internal Medicine, Scott and White Medical Center-Temple, Texas A&M Health Science Center, Temple, TX, United States

**Keywords:** hypertension, pregnancy, antihypertensive drugs, preeclampsia, cardiovascular

## Abstract

Treatment of pregnancy-related hypertensive disorders, such as preeclampsia (PE), remain a challenging problem in obstetrics. Typically, aggressive antihypertensive drug treatment options are avoided to prevent pharmacological-induced hypotension. Another major concern of administering antihypertensive drugs during pregnancy is possible adverse fetal outcome. In addition, management of hypertension during pregnancy in chronic hypertensive patients or in patients with prior kidney problems are carefully considered. Recent studies suggest that PE patients are at increased cardiovascular risk postpartum. Therefore, these patients need to be monitored postpartum for the subsequent development of other cardiovascular diseases. In this review article, we review the antihypertensive drugs currently being used to treat patients with PE and the advantages or disadvantages of using these drugs during pregnancy.

## Introduction

Preeclampsia (PE) is a clinical entity characterized by either the new onset of hypertension and proteinuria or end organ damage after 20 weeks of gestation. It is one of the major pregnancy-related hypertensive disorders and can occur postpartum. Additional clinical signs and symptoms include headache, visual disturbance, epigastric pain, thrombocytopenia, and abnormal liver function (1). These clinical manifestations are triggered by mild to severe microangiopathy of target organs, including the brain, liver, kidney, and placenta. Potential maternal complications include pulmonary edema, cerebral hemorrhage, hepatic failure, renal failure, and even death. Potential fetal complications are caused by placental hypoperfusion or the need for preterm delivery.

Traditionally, the clinical diagnosis of PE is made when new-onset hypertension in the second half of pregnancy is associated with new-onset proteinuria. However, following the observation that some patients show evidence of multiorgan damage without proteinuria, under certain circumstances PE can be diagnosed without proteinuria. In the absence of proteinuria, the diagnosis can be made if any of the following is present: abnormal liver function, thrombocytopenia, renal insufficiency, pulmonary edema, visual impairment, or cerebral symptoms. According to the 2013 report of the American College of Obstetricians and Gynecologists’ Task Force on Hypertension in Pregnancy, PE can be diagnosed when either (1) systolic blood pressure is greater than or equal to 140 mmHg or diastolic blood pressure is greater than or equal to 90 mmHg on two occasions at least 4 h apart in a previously normotensive patient or (2) systolic blood pressure is greater than or equal to 160 mm Hg or diastolic blood pressure is greater than or equal to 110 mmHg and hypertension can be confirmed within minutes to facilitate timely antihypertensive therapy. In addition to hypertension, proteinuria must be measured as greater than or equal to 300 mg per 24 h urine specimen, as a protein ratio greater than or equal to 0.3, or as a urine dipstick protein of 1+ (if a quantitative measurement is unavailable) (2).

Previously, PE was classified in terms of severity as mild, moderate, or severe. More recently, because morbidity and mortality can be significant for PE without severe features, the 2013 report of the American College of Obstetricians and Gynecologists’ Task Force on Hypertension in Pregnancy recommends that this classification be avoided (2). Instead, the term “preeclampsia without severe features” should be used to distinguish from more severe forms of “preeclampsia with severe features.” Based on the gestational age at delivery, PE has been broadly classified into early-onset, with signs and symptoms developing at <34 weeks of gestation, and late-onset in patient new-onset hypertension and proteinuria at ≥34 weeks of gestation, and sometimes during labor (Table 1). Although data is limited, it has been suggested that the maternal and perinatal mortalities varies in the subgroups of preeclampsia (3, 4). The early-onset PE consist of about 10% of total cases of PE and placental dysfunction is more likely to occur in this subgroup than in the more prevalent late-onset PE.

**Table 1:** Characteristics of the subgroups of preeclampsia.

PE Subgroup	Comment
Early onset PE(<34 weeks of gestation)	Consist of about 10% of total cases of PE.Placental dysfunction is more likely to occur; increase IUGR, maternal and perinatal mortalities.Renal function indicators (Cr, BUN and uric acid) were significantly higher, but alkaline phosphatase levels are lower, in early onset PE.
Late onset PE( ≥34 weeks of gestation/during labor)	Majority of cases of PE.Normal or big for gestational age fetus at delivery at term

BUN: blood urea nitrogen; Cr: serum creatinine; IUGR: intrauterine growth retardation; PE: preeclampsia.

PE may cause complications for patients with preexisting chronic hypertension or chronic kidney disease (CKD). Preexisting chronic hypertension is a principal risk factor for PE (5), and it usually portends a worse prognosis for the patient and the fetus. A diagnosis can be made when new-onset proteinuria and /or end-organ dysfunction occur after 20 weeks’ gestation in a woman with chronic/preexisting hypertension. For women with chronic/preexisting hypertension who have proteinuria prior to or in early pregnancy, a sudden exacerbation of hypertension or a need to increase antihypertensives, especially when blood pressure was previously controlled on these medications, will lead to a diagnosis of superimposed preeclampsia. The association of preeclampsia and later development of kidney disease is known. However, because CKD and PE may both present with hypertension and proteinuria in pregnancy, it is usually difficult to differentiate the two. Several efforts are being made to accurately distinguish CKD from PE, including using uteroplacental flows and maternal circulating soluble FMS-like tyrosine kinase-1 to placental growth factor ratio (6). To date, no curative treatment exists for PE; hence, the mainstay of management includes preventive measures for those at risk and, when PE occurs, stabilization of the mother and fetus followed by delivery at an optimal time (7).

## Management of Hypertension

Besides delivery of the fetus, the mainstay treatment for PE is control of hypertension with medication when indicated (Table 2). Various antihypertensive agents described below have been used in management of PE with severe features. However, a consensus has not been reached regarding the management of nonsevere hypertension. Salt restriction, bed rest, and restriction of physical activity is not recommended for prevention or treatment of PE without severe features (2).

**Table 2:** Commonly used antihypertensives in Preeclampsia with severe symptoms

Drugs	Indication	Dose	Comment
***First line***			
Methyldopa	PE with severe symptomsHypertension in pregnancy	0.5–3 g/day PO in 2 divided doses	Established long term safety.Breast milk compatibleMild hypertensive effect and slow onset of action, hence may not be used alone
Labetalol	PE with severe symptoms, usually IV formulation	Start with 20 mg IV bolusMayrequiredouble dose 10 min later	Rapid onset of actionStudies confirm safety in pregnancyMay cause maternal hepatotoxicity
Hydralazine	PE with severe symptoms, usually IV formulation.Long-acting nifedipine	5 mg IV slowly over 1 to 2 min30–90 mg once daily. May be increased at 7- to 14-day intervals, to maximum dose of 120 mg a day.	Usually breast milk compatible. More adverse effect than labetalolHypotensive effect is less predictable
Nefidipine	PE with severe symptoms, immediate release oral formulation	Start with 10 mg POMay repeat dose 30 min later	Use particularly when IV access is not available.May cause rapid drops in BP. Concern of serious side effects when used simultaneous with magnesium sulfate.
***Second line***			
Nicardipine	Resistant acute-onset severe hypertension when first line has failed	Give as IV infusion of 3 to 9 mg/hour	Delay onset of action (5–15 min). Titrate slowly to avoid overdose
Sodium Nitroprusside	Acute life threatening hypertension associated with PE	Start with 0.24 µg/kg/min.May titrate to maximum dose of 5 µg/kg/min	Rarely used in dire emergency.Give for shortest amount of time to avoid toxicity (cyanide & thiocyanate)

IV: intravenous; PE: preeclampsia; PO: per oral.

All antihypertensive medication can potentially cross the placenta. At this time, there are no randomized control trials to base a recommendation for the use of one antihypertensive agent over another. However, certain medications are effective in lowering blood pressure with an acceptable safety profile in pregnancy. The choice of therapy depends on the acuity and severity of hypertension. In addition, whether parenteral or oral therapy is preferred must be considered when choosing a particular drug.

Methyldopa stimulates the central alpha-adrenergic receptors by a false neurotransmitter (α-methylnorepinephrine), which results in a decreased sympathetic outflow of norepinephrine to the heart, kidneys, and peripheral vasculature. Methyldopa has been used extensively in the management of elevated blood pressure in pregnant women. In addition, its long-term safety for the fetus has been demonstrated. In the CHIPS (Control of Hypertension In Pregnancy Study) trial, preeclamptic women treated with methyldopa may have had better outcomes compared with those treated with labetalol (8). However, methyldopa has only a mild antihypertensive effect with a slow onset of action (3 to 6 h). Many preeclamptic women will not achieve blood pressure goals on this oral agent alone.

Labetalol lowers blood pressure by blocking β- and α-adrenergic receptors. In addition, it can better preserve uteroplacental blood flow compared with other β-blockers. It has a rapid onset of action (2 h) compared with methyldopa. Randomized clinical trials comparing labetalol to either methyldopa or nifedipine have shown labetalol to be safe for use in pregnancy (9, 10). Labetalol has been shown to cause maternal hepatotoxicity. It is important to recognize this side effect as it can be confused with HELLP (hemolysis, elevated liver enzymes, and low platelet count) syndrome. Most cases of labetalol induced hepatotoxicity are reversible, but fatalities have been reported (11).

Nifedipine is a calcium channel blocker that has been used in pregnancy without any major issue. Long-acting nifedipine is preferred over short-acting nifedipine as the short-acting version of the medication can cause a significant drop in blood pressure, possibly leading to a reduction in uteroplacental perfusion. However, as discussed below, more recent evidence suggest that immediate release oral nifedipine, under certain conditions, may be considered for safe reduction of BP. Long-acting nifedipine can be given as a sustained release tablet in doses of 30–90 mg once daily. The dosage can be increased at 7- to 14-day intervals, reaching a maximum dose of 120 mg a day (12).

Hydralazine is a direct vasodilator of arterioles. Intravenous hydralazine has been used extensively in the acute treatment of severe hypertension associated with preeclampsia. Although a meta-analysis showed a slightly increased rate of adverse events with hydralazine compared with labetalol, the evidence was not strong enough to recommend one medication over the other (13). However, the hypotensive effect of hydralazine is less predictable compared with other parenteral agents. The oral version of hydralazine can be used to treat hypertension associated with preeclampsia. However, it is limited by its side effects, including lower extremity swelling and reflex tachycardia.

As mentioned earlier, a consensus has not been reached regarding the management of PE without severe hypertension in patients who have chronic hypertension or CKD. For PE with severe hypertension (sustained systolic blood pressure of at least 160 mm Hg or diastolic blood pressure of at least 110 mmHg), the use of an antihypertensive is recommended (2). Labetalol, nifedipine, or methyldopa are recommended as first-line therapy. Relatively recent studies suggest that the calcium channel blocker, nifedipine, in its immediate oral release form also may be considered as a first-line therapy (14–18). The 2017 Committee Opinion by the American College of Obstetricians and Gynecologists’ Committee on Obstetric Practice recommended the use of immediate release oral nifedipine to be considered as a first-line therapy, particularly when IV access is not available. Renin-angiotensin-aldosterone system blockers—such as angiotensin-converting enzyme inhibitors, angiotensin receptor blockers, renin inhibitors, and mineralocorticoid receptor antagonists—should be avoided. The recommended blood pressure measurements are between 120/80 mm Hg and 160/105 mm Hg.

It is well known that antihypertensive agents do not prevent eclampsia (seizure). Although magnesium sulfate is not recommended as an antihypertensive agent, it has been used over the years for the prevention of seizure in preeclamptic women with severe features and for managing recurrent seizures in eclampsia. However, limited double-blind, placebo-controlled trials have shown that there is no a significant difference in occurrence of eclampsia between patients with pre-eclampsia without severe features treated with magnesium sulfate and those given placebo (19). The American College of Obstetricians and Gynecologists’ (ACOG) in 2013 recomendations, states that “for women with preeclampsia with systolic blood pressure of less than 160 mmHg and a diastolic blood pressure less than 110 mmHg and no maternal symptoms, it is suggested that magnesium sulfate not be administered universally for the prevention of eclampsia”. In other words the ACOG limits use of magnesium sulfate for prevention of eclampsia to patients with BP of 160/110 or higher, or if blood pressure is less than 160/110, other severe symptoms that usually precede seizures are present. Studies have shown that magnesium sulfate is more effective for prevention of recurrent seizures in eclampsia than other traditional anticonvulsants including phenytoin and diazepam, or lytic cocktails (20–22). The mechanism of action of magnesium sulfate in preventing seizure is not completely understood but it is thought to be due to its effect on the central nervous system, possibly due to its effect on the n-methyl d-aspartate (NMDA) receptors, calcium channels, and acetylcholine. Although data is limited, the concern that simultaneous use of magnesium sulfate and nefidipine will lead to serious side effects including hypotension and neuromuscular inhibition was not supported by available evidence (23).

## Conclusions

When treatment of hypertension in PE is indicated, management generally depends on the acuity and severity of the hypertension as well as comorbidities. The choice of agents, for the most part, depends on availability of an agent and the experience of the provider. Some authorities have recommended labetalol coupled with calcium channel blockers as first-line drugs in PE patients with chronic hypertension, further suggesting these agents be maxed out before starting another agent. In acute-onset severe hypertension, it is recommended to give intravenous labetalol, hydralazine, or oral nifedipine as first-line agents

Women with a history of PE experience a 2-fold increased risk of long-term cardiovascular disease and an approximate 5- to 12-fold increased risk of end-stage renal disease. Recognizing PE as a risk factor for future renal and cardiovascular disease will allow for identification of a population of young women who at high risk of developing cardiovascular and renal disease. The current guidelines recommend cardiovascular screening and treatment for formerly preeclamptic women. However, because of a lack of studies on screening and prevention in formerly preeclamptic women, these recommendations are based on low levels of evidence. Hence, research is needed on mechanisms of late disease manifestations and on effective screening and therapeutic strategies aimed at reducing the late disease burden in formerly preeclamptic women (24–26).

## Author Contributions

OO, LJP, PC conceived, wrote sections of the manuscript, revised the first draft, and finalized the manuscript. All authors contributed to manuscript revision as well as read and approved the submitted version.

## Conflict of Interest Statement

The authors declare that the research was conducted in the absence of any commercial or financial relationships that could be construed as a potential conflict of interest.

The reviewer MC and handling Editor declared their shared affiliation.
